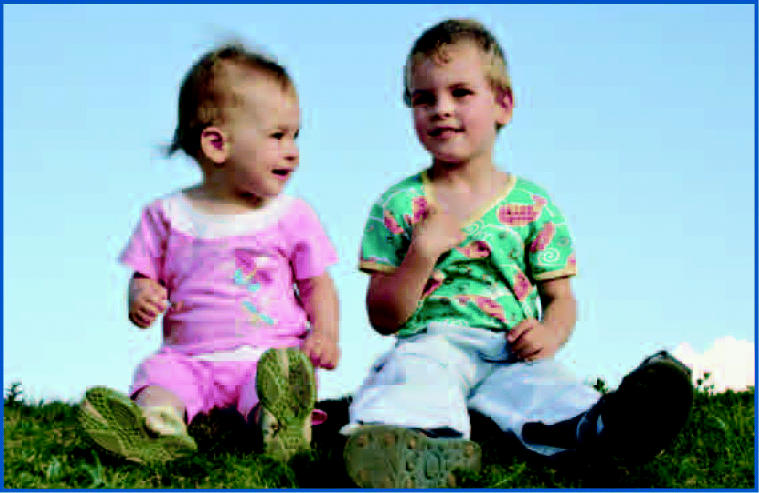# Headliners: Respiratory Health: Effects in Infants from Tobacco Smoke, Mold, and Older Siblings

**Published:** 2006-10

**Authors:** Jerry Phelps

Biagini JM, LeMasters GK, Ryan PH, Levin L, Reponen T, Bernstein DI, et al. 2006. Environmental risk factors of rhinitis in early infancy. Pediatr Allergy Immunol 17(4):278–284.

Many environmental exposures have been confirmed to affect children’s respiratory health, but few have been studied in very young children. Now NIEHS grantees Grace K. LeMasters, Jocelyn M. Biagini, and their colleagues at the University of Cincinnati and the Cincinnati Children’s Hospital Medical Center demonstrate for the first time the relationship between exposure to environmental tobacco smoke (ETS) and allergy in infants.

About one-fifth of all American adults smoke cigarettes, resulting in about 43% of children being exposed to ETS at home. ETS exposure, along with mold exposure, has been documented as a risk factor for health problems such as wheezing, asthma, and otitis media in both children and adults.

In the current study, the researchers observed the effects of ETS and indoor mold exposure on the development of rhinitis and symptoms such as nasal blockage, sneezing, and nasal itching in a cohort of 633 infants under the age of 1 enrolled in the Cincinnati Childhood Allergen and Air Pollution Study. They used interviewer-administered questionnaires to collect demographics and information on smoking habits, family health history, and other covariates. They also analyzed any upper respiratory symptoms of the infants recorded by the parents in a monthly diary. In addition, they performed a skin-prick test on the parents and the infants (at approximately 12 months of age) to test for sensitivity to at least 1 of 15 airborne allergens.

The investigators found that exposure to ETS increased an infant’s risk of developing allergic rhinitis by almost threefold. They also found that exposure to mold in the home was associated with increased risk of upper respiratory infections but not allergy, which differed from previously reported research in older children and adults.

Other findings included a protective effect of having older siblings in the home. Infants with at least one older sibling were less likely to have allergic rhinitis by their first birthday. This finding supports the hygiene hypothesis, a theory that exposure to infectious agents early in life may decrease the risk for allergic diseases such as asthma later in life. Presumably, by having older siblings these infants were exposed to a wider variety of viruses and bacteria, causing their immune systems to develop in a way that decreased the risk of allergy.

The authors conclude that further research is necessary to confirm their results. Continued research is also needed to determine the components of cigarette smoke that cause these health effects, and to ascertain the role of possible gene–environment interactions.

## Figures and Tables

**Figure:**